# 

**Published:** 1908-05-02

**Authors:** 


CONTENTS.

| | PAGE |
|---|---|
| **The Research Defence Society...** | 109 |
| **Superfluous Medical Literature** | 110 |
| **Annotations—** | |
| Electro-Therapeutics—Doctors' Bills—Death of Professor von Schrötter | 111 |
| **Medical Opinion and Movement** | 112 |
| **Hospital Clinics—** | |
| The Indications and Contra-Indications for Curetting the Uterus. By J. Inglis Parsons, M.D., M.R.C.P.... | 115 |
| **Special Article—** | |
| The Amblyopia Produced by Certain Drugs | 116 |
| **Medicine—** | |
| Sudden Death in Pleural Effusion Cases | 119 |
| **Illustrative Cases—** | |
| Hemiplegia from Tuberculous Meningitis | 120 |
| **Notes on Treatment** | 120 |
| **Points in Surgery—** | |
| Aneurysm | 121 |
| **Diseases of Children—** | |
| Polyuria | 122 |
| **Tropical Diseases—** | |
| Filariasis—I. | 123 |
| **Mental Diseases—** | |
| Early and Mild Cases of Mental Disorder | 124 |
| **Laryngology and Rhinology—** | |
| Syphilis of the Throat and Nose—I. | 125 |
| **The Royal Army Medical Corps Section—** | |
| Territorial Cavalry Ambulance ... | 126 |
| **Pathology—** | |
| The Eosinophilia of Hydatid Disease... | 127 |
| **Public Health and Hygiene—** | |
| Diagram of the Weekly Death Rate in 1908... | 128 |
| **Hospital Administration—** | |
| Hampstead General Hospital | 129 |
| Graduate Study on the Continent—II. | 131 |
| Research Defence Society... | 132 |
| **Nursing Administration—** | |
| The Bedding and Linen Department ... | 133 |
| **Editor's Letter-Box** | 134 |
| **Reviews...** | 135 |
| **The Graduates' Medical Schools** | 136 |

